# Spatio-Temporal Dynamics of Tick-Borne Diseases in North-Central Wisconsin from 2000–2016

**DOI:** 10.3390/ijerph17145105

**Published:** 2020-07-15

**Authors:** Austin Rau, Claudia Munoz-Zanzi, Anna M. Schotthoefer, Jonathan D. Oliver, Jesse D. Berman

**Affiliations:** 1 Division of Environmental Health Sciences, School of Public Health, University of Minnesota, Minneapolis, MN 55455, USA; rauxx087@umn.edu (A.R.); munozzan@umn.edu (C.M.-Z.); joliver@umn.edu (J.D.O.); 2 Marshfield Clinic Research Institute, Marshfield, WI 54449, USA; schotthoefer.anna@marshfieldresearch.org

**Keywords:** spatial epidemiology, geographic information systems (GIS), SatScan, spatial analysis, tick-borne diseases

## Abstract

Lyme disease is a well-recognized public health problem in the USA, however, other tick-borne diseases also have major public health impacts. Yet, limited research has evaluated changes in the spatial and temporal patterns of non-Lyme tick-borne diseases within endemic regions. Using laboratory data from a large healthcare system in north-central Wisconsin from 2000–2016, we applied a Kulldorf’s scan statistic to analyze spatial, temporal and seasonal clusters of laboratory-positive cases of human granulocytic anaplasmosis (HGA), babesiosis, and ehrlichiosis at the county level. Older males were identified as the subpopulation at greatest risk for non-Lyme tick-borne diseases and we observed a statistically significant spatial and temporal clustering of cases (*p* < 0.05). HGA risk shifted from west to east over time (2000–2016) with a relative risk (RR) ranging from 3.30 to 11.85, whereas babesiosis risk shifted from south to north and west over time (2004–2016) with an RR ranging from 4.33 to 4.81. Our study highlights the occurrence of non-Lyme tick-borne diseases, and identifies at-risk subpopulations and shifting spatial and temporal heterogeneities in disease risk. Our findings can be used by healthcare providers and public health practitioners to increase public awareness and improve case detection.

## 1. Introduction

Tick-borne diseases pose a significant public health challenge to the USA with evidence of increasing occurrence in the last decade. Between 2016 and 2018 alone, there were 155,702 reported cases of tick-borne diseases in the USA, of which 112,838 (72.5%) were identified as Lyme disease cases. Although Lyme disease is the major leading cause of tick-borne disease in the USA and has been extensively studied, the three next leading causes of tick-borne disease (anaplasmosis/ehrlichiosis, Rocky Mountain spotted fever and babesiosis) accounted for 42,090 cases (37.3%) in 2016–2018 [1]. However, these diseases have received less attention compared to Lyme disease. The effects of tick-borne diseases such as human granulocytic anaplasmosis (HGA) and babesiosis can be serious and, in some cases, become life threatening [2,3], which underscores the need to study non-Lyme tick-borne diseases to prevent future morbidity and mortality in the population. Specifically, non-Lyme tick-borne diseases are of concern for states in the upper Midwest including Minnesota and Wisconsin, an endemic region for non-Lyme tick-borne diseases with a high incidence of HGA and babesiosis [4]. However, there is limited research on human non-Lyme tick-borne disease cases in this region. We addressed this gap in our understanding by investigating changes in the risk of non-Lyme tick-borne diseases in a large health care population in Wisconsin.

Past research described the geographical spread of *Ixodes scapularis* (the vector of the causative agents for Lyme disease, HGA, babesiosis and a strain of recently identified *Ehrlichia*) in Wisconsin from the western part of the state beginning in the 1980s and continuing into the 2000s [5,6,7]. Additionally, several studies described the prevalence of infection in *I. scapularis* ticks collected at sites across Wisconsin [8,9,10]. Indeed, virtually all of Wisconsin is currently classified as having evidence of established *I. scapularis* populations and having high predicted acarological risk for Lyme disease [11,12]. Other studies in neighboring Minnesota examined habitat suitability and environmental predictors of *I. scapularis* and diseases vectored by the organism [13,14,15]. While specific causes of the increased geographic expansion of *I. scapularis* are unknown, reforestation, increasing abundance of white-tailed deer and climate change are proposed contributing factors [16]. However, despite an abundance of research investigating changes in *I. scapularis* populations, there remains a paucity of research investigating the trends and spatial distributions of human cases of tick-borne diseases, particularly non-Lyme tick-borne diseases. We still know little about how the risk of non-Lyme tick-borne diseases has changed over space and time in the upper Midwest.

As the distribution of *I. scapularis* changed over time in Wisconsin, we hypothesized the risk of non-Lyme tick-borne diseases was not stationary in space or time within a large healthcare population in north and central Wisconsin. To test our hypothesis, we applied a Kulldorf’s scan statistic to evaluate changes in risk over space and time among laboratory-positive cases of three tick-borne diseases, HGA, babesiosis and ehrlichiosis (heretofore referred to as non-Lyme tick-borne disease) recorded in the Marshfield Clinic Health Care System (MCHS) from 2000 to 2016. Reservoirs for these diseases include the white-footed mouse and other small mammals for *A. phagocytophilum*, the white-footed mouse for *B. microti* and the white-tailed deer for *E. chaffeensis* and *E. ewingii* [16]. The current reservoir for *E. muris eauclairensis* is unknown due to the pathogen being recently discovered [16,17]. The results of this study will elucidate geographical areas and moments in time of increased risk for non-Lyme tick-borne diseases. Our findings may aid in the identification of at-risk populations for non-Lyme tick-borne diseases and help clinicians and public health experts prepare for temporal fluctuations in cases.

## 2. Materials and Methods

### 2.1. Study Population

**Figure 1:**
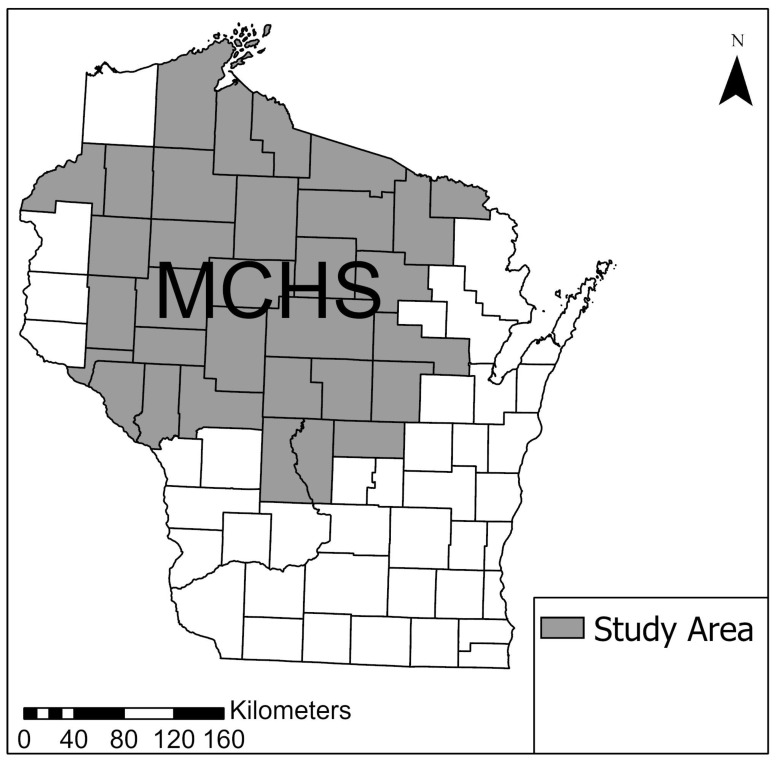
The Marshfield Clinic Health System (MCHS) study area located in the central and northwestern portion of Wisconsin (denoted in gray)**.**

We obtained electronic health records (EHRs) of patients who received medical care within the MCHS between 2000–2016 to calculate rates of infection for non-Lyme tick-borne diseases. The MCHS serves a population of about 700,000 people living primarily in northern and central Wisconsin (Figure 1), a region with high risk for tick-borne diseases [18]. Monthly and yearly counts of both total users and non-Lyme tick-borne disease cases within the MCHS system, stratified by age and sex of the patient population, were obtained from the EHRs and used to estimate disease rates. The population of the MCHS was relatively stable over time (Figure S1) and includes a cohort of about 185,000 patients who live in a 24 zip-code region within the service area and receive the majority of their outpatient care at a Marshfield associated clinic facility [19].

### 2.2. Data Confidentiality

Approval to use patient health information in this study was obtained from the Institutional Review Boards (IRB) at the Marshfield Clinic Research Institute (Marshfield, WI, USA, SP Code: SCH10110) and the University of Minnesota Twin Cities (Minneapolis, MN, USA, Protocol ID: 1605M87224).

### 2.3. Data Inclusion

#### 2.3.1. Case Identification

We identified records of patients with laboratory evidence of non-Lyme tick-borne diseases based on MCHS EHRs laboratory codes for HGA, babesiosis and ehrlichiosis derived from blood smear, serologic, and PCR tests completed between 2000–2016. The MCHS includes Marshfield Labs, a central laboratory serving the health care providers and patient population throughout the service area. MCHS recommends providers use the laboratory tests following national guidelines for diagnosis of HGA, babesiosis and ehrlichiosis [20,21,22], though it allows providers to use their clinical discretion in ordering tests. Between 2000 and 2013, each test type for each disease had to be ordered separately, beginning in 2013, an option to order the PCR tests for HGA, babesiosis and ehrlichiosis using a single test code, became available. Tests are available year-round. Of the 50,575 records identified, 3682 were associated with laboratory-positive test results. We applied the laboratory criteria in the 2008 Council of State and Territorial Epidemiologists (CSTE) case definition for HGA and ehrlichiosis [23]. HGA included records with supportive laboratory criteria for diagnosis (positive blood smear or indirect immunofluorescence titer (IFA) ≥1:64) and records with confirmed criteria (positive PCR or 4-fold change in titers in paired serum). Similarly, ehrlichiosis (caused by *E. chaffeensis*) included records with supportive criteria for diagnosis (positive blood smear or IFA titer ≥1:64) and records with confirmed criteria (positive PCR). Ehrlichiosis caused by either *E. ewingii* or *E. muris eauclairensis* included records with confirmed criteria (positive PCR). Given the small sample size of ehrlichiosis cases (*n* = 30) and to narrow the focus to a common vector for the pathogens under study (*I. scapularis*), we only considered cases of ehrlichiosis caused by *E. muris eauclairensis* (*n* = 15) endemic to Wisconsin and recently described in humans [17,24]. We used the 2011 CSTE case definition for babesiosis [25] which included records with supportive laboratory criteria (IFA titer ≥1:256) and with confirmed criteria (positive PCR or positive blood smear) for babesiosis caused by *B. microti*.

If patients had tests completed on more than one date within a 30-day period (*n* = 198), we combined the results of the tests into a single record associated with the earliest test positive date. Positive PCR or blood smear tests performed more than one year apart on the same patient were treated as evidence of separate infection events. However, since antibodies for HGA and babesiosis are known to persist for some time following infections [3,26,27] we assumed if a patient had more than one positive serologic test result for a specific disease within a one year period (*n* = 322) it was associated with the same illness event and only the earliest test date records were retained. 

For patients with positive serologic test dates approximately two years apart (*n* = 81), patient records were manually reviewed for acute symptoms (e.g., fever, headache, myalgias) and evidence of tick exposure (e.g., patient recorded a tick bite or engaging in an activity which could lead to tick exposure) around each test date to determine evidence of reinfection. If evidence of reinfection existed, both records were retained, but if the persistence of antibodies was a more likely explanation, only the record associated with the first positive test event was retained. Finally, for patients with positive test dates greater than two years apart for a specific disease (*n* = 151), it was assumed the dates were associated with separate infection events and records were retained in the database. 

#### 2.3.2. Geocoding Patients

A systematic approach was applied to geocode patients’ home locations provided in the MCHS EHR data (Figure S2). Home addresses at the date of laboratory testing were geocoded to the point address or street level using ArcGIS World Geocoding Services in ArcGIS Pro (ESRI ArcGIS Pro: Version 2.3.0. Redlands, CA, USA) [28]. We manually investigated four records whose geocoded address match score was below 80% (i.e., the address found by ArcGIS Pro did not sufficiently match the recorded address of a patient) and used Google Maps to manually update addresses. While most patients had a complete address provided, 34 had no address listed and 105 only had a Post Office (PO) box number provided; zip code and county of residence information were available for all records in the dataset. 

If a patient’s home address could not be geocoded, we matched the record’s zip code to the Zip Code Tabulation Area (ZCTA) numbers in Wisconsin. For patients whose laboratory test date was between 2000 to 2009, the centroid of the year 2000 ZCTA boundaries were used as their spatial locations. For patients whose laboratory test date was between 2010 to 2016, the centroid of the 2010 ZCTA boundaries were applied. Matching on ZCTA failed for two patients and we assigned them the county of residence centroid provided in the EHRs. We did not include patients whose address or county of residence was outside the state of Wisconsin. The total number of cases per county was represented as the sum of all patients aggregated to the county boundaries (Figure S2). 

### 2.4. Statistical Analysis

The general approach for analysis consisted of several parts: we performed descriptive summaries of cases by disease and demographics, spatial cluster analysis of disease for the entire study period and over four distinct time periods, temporal cluster analysis and seasonal cluster analysis of disease. The statistical methods used to identify spatial, temporal and seasonal clusters of positive laboratory results for non-Lyme tick-borne diseases reported the relative risk of having a positive laboratory result for a non-Lyme tick-borne disease. In the case of spatial clusters, the relative risk is the risk of having a positive laboratory result for a non-Lyme tick-borne disease when residing within a geographic cluster versus residing outside of a geographic cluster. In the case of temporal and seasonal clusters, the relative risk signified the risk of a positive laboratory result for a non-Lyme tick-borne disease within a specific window of time compared to the time outside of that window of time. 

#### 2.4.1. Descriptive Statistics

Summary statistics on the number of non-Lyme tick-borne disease cases by disease type were calculated, stratifying age and sex separately. We created three age groups to describe differences in laboratory testing and disease prevalence for young (<15 years), middle (15–64 years) and older (65+ years) patients. Temporal trends in the incidence rates of non-Lyme tick-borne diseases over time were plotted. For analysis purposes, we combined cases with laboratory confirmatory and laboratory supportive evidence due to limited sample size. In addition, the number of cases by season (where season is the number of cases by month irrespective of the year in which cases occurred) were plotted along with the geographic changes in incidence rates over time at the county level. Incidence rates for non-Lyme tick-borne disease were calculated using the MCHS population data as the population at-risk. All exploratory data analysis was completed using R statistical software (version 3.6.0, R Foundation for Statistical Computing, Vienna, Austria) [29]. 

#### 2.4.2. SatScan Analysis

We used Kulldorf’s spatial scan statistic software, SatScan (Kulldorf M. and Information Management Services, Inc. SaTScan™ v9.6: Calverton, MD, USA) [30], to analyze retrospective spatial, temporal and seasonal clustering of cases by disease type using a discrete Poisson model [31]. Past research utilized SatScan to analyze the spatial and temporal dynamics of Lyme disease and other tick-borne diseases [32,33,34,35,36,37]. The scan statistic tests the hypothesis of an elevated risk of non-Lyme tick-borne disease within a window (spatial or temporal) versus outside of the window. Significance of the cluster is based upon Monte Carlo simulation, which compares the observed number of cases within a cluster to the expected number in that cluster under the null hypothesis [38]. Spatial clusters are additionally ranked from most to least likely with a likelihood ratio statistic generated by SatScan.

#### 2.4.3. Spatial Cluster Analysis

Spatial cluster analysis was performed for HGA and babesiosis separately and for all three diseases combined for the entire study period 2000–2016. In addition, spatial cluster analysis for HGA and babesiosis was executed for four distinct time periods (2000–2003, 2004–2007, 2008–2011, and 2012–2016) to examine changes in the location of spatial clusters over time. We selected multi-year time intervals, as opposed to individual years, to capture windows of time that were not too narrow (i.e., there would not be enough cases) nr too broad (i.e., we would miss the changes in spatial patterns over time). Maps of the spatial clusters were created in QGIS (3.8.0, Open Source Geospatial Foundation Project version) [39]. Ehrlichiosis cases (caused by *E. muris eauclairensis*) were not analyzed separately due to low case numbers (*n* = 15).

The spatial scan statistic operates by applying an elliptical or circular window on the study area which is centered upon each county centroid in our study area. For our spatial scan analysis, ellipses were used to capture anisotropy in clusters [38] and a medium penalty score was applied to favor more compact versus geographically spread clusters [38]. The elliptical window radius varied from a size of 0 percent of the population at risk to an upper limit based on county population at risk allowing for clusters to vary in location and size [38]. In our study, the population at risk was the monthly population count of users in the MCHS for each county and the upper limit for the spatial cluster radius chosen was 50% of the total MCHS population. This approach accounts for spatial heterogeneities in the at-risk populations as well as the utilization of MCHS for health care across counties in the MCHS service area [38]. A total of 999 permutations were completed for all spatial analyses, an α level of 0.05 was used to determine statistical significance, and we reported the most likely spatial cluster based on hierarchical rankings. 

#### 2.4.4. Temporal and Seasonal Cluster Analysis

We completed a separate temporal cluster analysis to evaluate time variance of disease using a monthly interval for HGA and babesiosis from 2000–2016. The temporal cluster analysis can identify moments in time of elevated risk for HGA and babesiosis along a linear time axis starting on 1 January 2000 and ending on 31 December 2016. We examined the sensitivity of the temporal window selection by iteratively modifying window sizes at 50, 40, 30, 20 and 10% of the total study time period for the temporal analyses. 

In addition, a seasonal cluster analysis was completed with a maximum cluster window of up to 50% of the total number of months in a year (6 months total) to identify months of the year that had a higher risk of HGA and babesiosis laboratory positive test results. The seasonal cluster analysis allows for the identification of months of elevated risk regardless of the specific year in which cases occurred [38], different from the temporal cluster analysis which identifies clusters with respect to year of occurrence for the cases. A total of 999 permutations were completed for all temporal analyses and an α level of 0.05 was used to determine statistical significance.

## 3. Results

### 3.1. Descriptive Statistics

After applying the study case definitions to the original 3682 laboratory positive results, the final geocoded dataset of non-Lyme tick-borne disease cases occurring between 2000–2016 in the MCHS was 2956 (laboratory-confirmed and supportive test results from individual patients). HGA represented the majority of cases: 2728 out of 2956 (92.3%)), of which 809 (29.7%) were laboratory-confirmed (665 (82.2%) were PCR-positive and 144 (17.8%) had evidence of a 4-fold change in IFA titers in paired serum) and 1919 (70.3%) of HGA cases had laboratory supportive evidence (109 (6%) were smear-positive and 1810 (94%) had single IFA titers ≥1:64) (Table 1). Babesiosis accounted for 213 cases (7.2%), of which 98 (46%) were laboratory confirmed (55 (56.1%) were PCR-positive, 26 (26.5%) were smear-positive and 17 (17.4%) were both smear- and PCR-positive) and 115 cases (54%) were laboratory supportive (IFA titers ≥1:256) (Table 1). Ehrlichiosis represented the smallest number of cases, with 15 (<1%), all of which were laboratory-confirmed (PCR-positive). Of the 2956 cases, 28 cases (<1%) were co-infections of more than one pathogen, all of which were HGA and babesiosis co-infections. Two cases (7.1%) were laboratory-confirmed for both HGA and babesiosis, 1 case (3.6%) was laboratory-confirmed for HGA and laboratory-supportive for babesiosis, 11 cases (39.3%) were laboratory-confirmed for babesiosis and laboratory-supportive for HGA, and, finally, 14 cases (50%) were laboratory-supportive for both HGA and babesiosis. 

For each of the three diseases, the mean age of cases (laboratory-confirmed and -supportive combined) was over 50 years. The mean age of HGA cases was 54 years. One-hundred-and-forty cases (5.1%) were in patients under 15 years of age, 1710 cases (62.7%) were in patients aged 15 to 64 and 878 cases (32.2%) were in patients over age 65. The mean age of babesiosis cases was 59 years with two cases (<1%) under age 15, 129 cases (60.6%) between the ages of 15 to 64 and 82 cases (38.5%) over age 65. Ehrlichiosis cases affected older patients with a mean age of 69 years and range of 60 to 83 years. HGA and babesiosis cases spanned a wide range of ages from young children to elderly adults (Table 1). Cases were normally distributed by age, with similar mean and median values. The mean age of cases for all three diseases was variable across the study period (Figure S3). Babesiosis cases, on average, for males and females tended to be between 50 to 70 years from 2000–2016. In 2008, there was a marked decrease in the mean age of female cases with an average of 30 years old; however, there were few female babesiosis cases in that year. The mean age of HGA cases for males and females fluctuated between 45 to 60 years from 2000–2016. Male ehrlichiosis cases tended to be older, on average, compared to females in 2015–2016, yet females were typically older, on average, than males in 2013–2014 (Figure S3). Examination of the distribution of positive laboratory tests by sex for HGA and babesiosis indicated that males were the majority of cases for all test types (Table 1). Laboratory-confirmed cases for HGA and babesiosis tended to be older (mean of 59 years for HGA and babesiosis) compared to laboratory-supportive cases (mean of 51 years for HGA and 56 years for babesiosis). The average age of cases tended to be younger for serologic tests compared to cases diagnosed using PCR or smears for HGA and babesiosis (Table 1).

### 3.2. Temporal and Seasonal Trends

**Figure 2:**
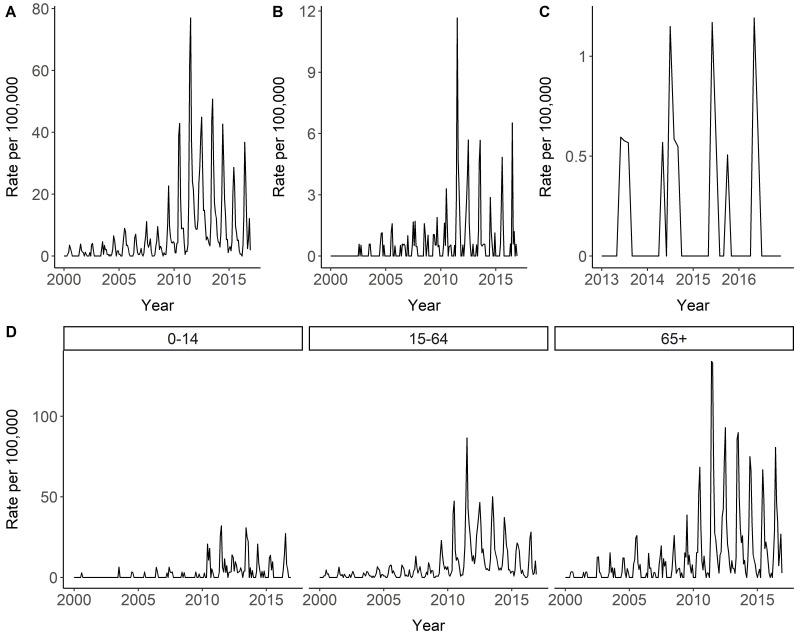
Positive laboratory cases in the Marshfield Clinic Health System for human granulocytic anaplasmosis (HGA), babesiosis, and ehrlichiosis over time and stratified by age (0–14 years, 15–64 years, ≥65 years) from 2000–2016. (**A**) displays monthly incidence rate of laboratory positive cases of HGA per 100,000 patients; Panel (**B**) displays monthly incidence rate of laboratory positive cases of babesiosis per 100,000 patients; Panel (**C**) displays monthly incidence rate of laboratory positive cases of ehrlichiosis per 100,000 patients; (**D**) displays monthly incidence rate of laboratory positive cases (all diseases combined) stratified by broad age categories per 100,000 patients.

Temporal trends in laboratory-positive cases were similar for HGA and babesiosis with the monthly incidence rate of disease being low in the early part of the study period and steadily increasing during the latter half of the study period in the MCHS (Figure 2A,B). For instance, between 2000–2005, the maximum monthly incidence rates did not exceed nine per 100,000 for HGA and two per 100,000 for babesiosis. A spike in incidence rates was observed in 2011 for both HGA and babesiosis compared to the prior year. For comparison, in 2011, the maximum monthly rates increased to 77 per 100,000 for HGA, compared to 2010 (a greater than 3-fold rise compared to 2010) and 11.7 per 100,000 for babesiosis (a greater than 6-fold rise compared to 2010). Incidence rates remained high for HGA during the remainder of the study period post-2011. Ehrlichiosis cases due to *E. muris eauclairensis* were not detected in the MCHS until 2013 and few cases were identified from 2013 to 2016 (Figure 2C). Patients over age 65 had the highest incidence rates overall and patients aged 0–14 had the lowest incidence rates (Figure 2D). All age groups experienced a large spike in incidence rates in 2011.

**Figure 3:**
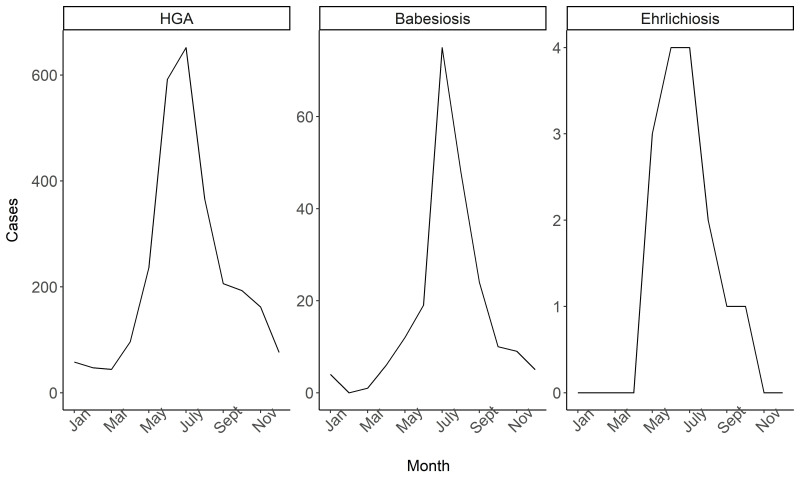
Counts of laboratory-positive non-Lyme tick-borne disease cases in the Marshfield Clinic Health System (MCHS) by disease type from 2000–2016 stratified by month.

Seasonal trends in laboratory positive cases for all diseases indicated summer months (June–August) had the greatest number of cases, whereas winter months (December–February) had the lowest number of cases (Figure 3). HGA cases were detected year-round but increased starting in April (96 cases) to May (236 cases). Specifically, HGA cases peaked in July (652 cases) and had high numbers of cases throughout the summer. While cases of babesiosis were detected between January to March, only five cases occurred in this timeframe. Babesiosis cases started to ramp up in May (12 cases) and peaked in July (75 cases) with a rapid increase from June (19 cases) to July (75 cases), a 295% increase in cases, which fell to 48 cases in August and continued to decline throughout the remainder of the year. The majority of ehrlichiosis cases were detected in May–August (13 cases) and cases peaked in June and July with four cases in both months. No ehrlichiosis cases were detected prior to May or after October.

For all diseases combined, summer months (June–August) represented the most cases for all ages (age 0–14: 90 out of 142 cases (63.4%), age 15–64: 1012 out of 1789 cases (56.6%), age 65+: 660 out of 1025 cases (64.4%)). Patients over age 65 experienced the most cases in July, with 271 cases (26.4%) of the total 1025 cases occurring in that month. Patients aged 15–64 also experienced the greatest number of cases in July with 427 (23.9%) of the 1789 total cases occurring in that month. Finally, patients aged 0–14 experienced the greatest number of cases in June, with 34 (23.9%) of the 142 total cases occurring in that month. Seasonal trends by sex revealed that July had the greatest number of cases for males (421 out of 1620 cases (26%)) and for females (310 out of 1336 cases (23.2%)).

### 3.3. Spatial Patterns and Trends Over Time

**Figure 4:**
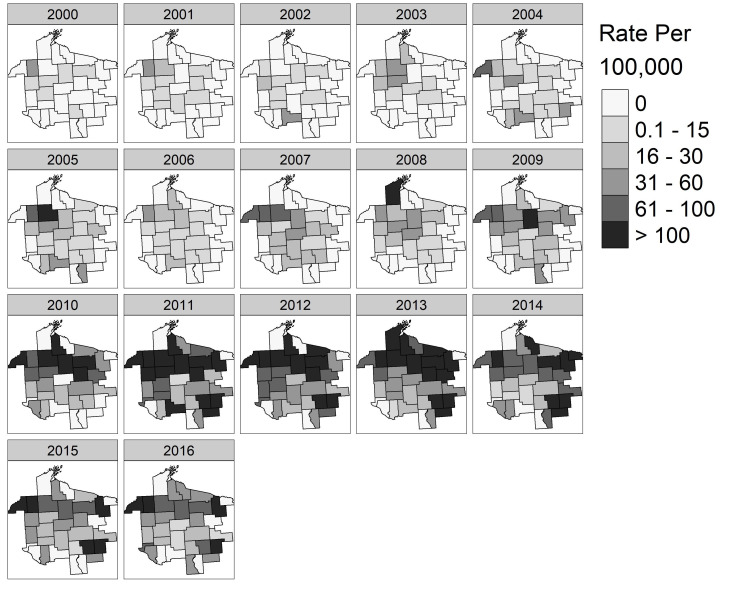
Annual county level incidence rate for positive laboratory cases of combined tick-borne diseases caused by human granulocytic anaplasmosis, babesiosis, ehrlichiosis per 100,000 patients, 2000–2016 for the Marshfield Clinic Health System.

The spatial distribution of HGA, babesiosis and ehrlichiosis combined revealed increasing incidence rates in the MCHS over time with a shifting geographic area of risk. Incidence rates in 2000–2004 were low, with many counties in the MCHS having zero cases. Incidence rates steadily increased between 2005–2010 with counties in the north central region of the MCHS having higher incidence rates than the rest of the study area. Incidence rates above 100 per 100,000 dominated the MCHS between 2011–2013 in the north central region, shifting eastward in 2013 and 2014. Incidence rates decreased in 2015 and 2016 in the north-central and eastern regions of the MCHS, yet most counties remained classified as having 31–100 cases per 100,000 (Figure 4).

### 3.4. Spatial Cluster Analysis

**Figure 5:**
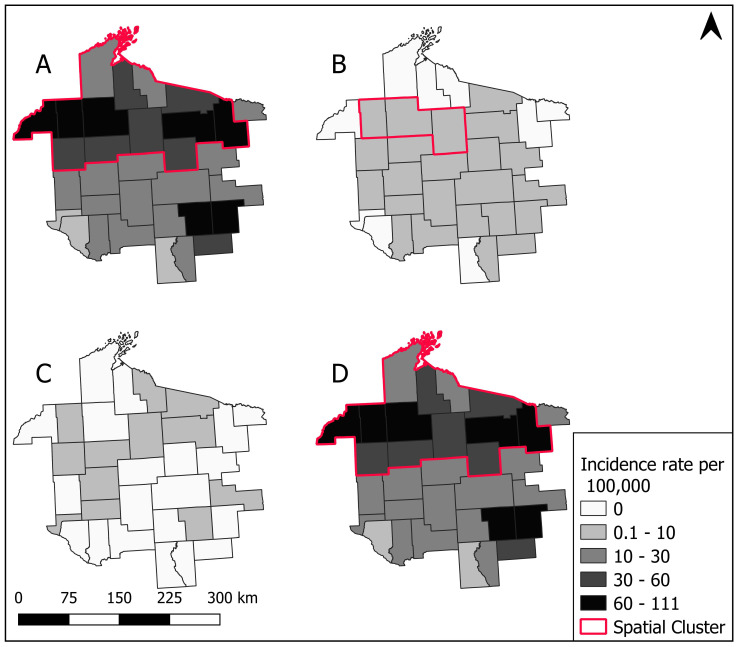
County level incidence for positive laboratory cases of (**A**) human granulocytic anaplasmosis (HGA), (**B**) babesiosis, (**C**) ehrlichiosis, and (**D**) all diseases combined per 100,000 patients in 2000–2016. The red line denotes primary spatial clusters. Spatial clusters were not calculated for ehrlichiosis due to small numbers of cases (*n* = 15).

The primary spatial cluster for HGA (2000–2016) accounted for 53.30% of all HGA cases and the risk of having a positive HGA laboratory test result was three times greater for patients residing within the cluster versus outside of the cluster. The primary spatial cluster for HGA was located in the northern half of the MCHS (Figure 5, Table 2). The primary spatial cluster for babesiosis (2000–2016) occupied a small geographic area in the northwestern part of the MCHS, with patients residing within the cluster having a 3.9 times greater risk of a positive babesiosis laboratory test result than patients residing outside of the cluster (Figure 5, Table 2). The primary spatial cluster accounted for 17.84% of all babesiosis cases. Finally, for all three diseases combined, the primary spatial cluster had the same spatial distribution as the HGA primary spatial cluster, yet the relative risk of a positive laboratory result for a non-Lyme tick-borne disease was 2.92 for within the cluster compared to outside the cluster. In each of the spatial cluster analysis assessments for the entire study time period, the spatial scanning windows for SatScan never exceeded 28% of the population at risk.

**Figure 6:**
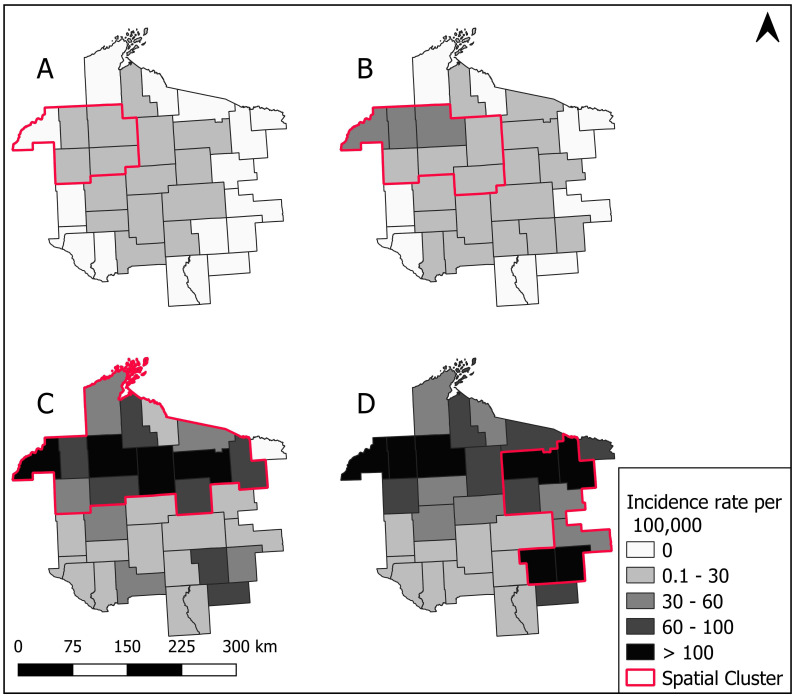
County level incidence of positive laboratory cases of human granulocytic anaplasmosis (HGA) per 100,000 patients from time periods of (**A**) 2000–2003, (**B**) 2004–2007, (**C**) 2008–2011, (**D**) 2012–2016 in the Marshfield Clinic Health System. The respective primary spatial clusters are outlined in red.

We additionally evaluated changes in the location and magnitude of risk in spatial clusters over time by completing a spatial SatScan analysis of HGA and babesiosis for four separate timeframes (2000–2003, 2004–2007, 2008–2011 and 2012–2016) in which we estimated the most likely spatial cluster for each time frame. For HGA, the primary spatial cluster from 2000–2003 was located in the northwest portion of the MCHS and had a relative risk of 11.85. Of the 79 total HGA cases which occurred during 2000–2003, 63.30% of the cases occurred within the cluster (Figure 6, Table 2). In 2004–2007, the primary spatial cluster expanded to encompass a larger swath of territory in the northwestern part of the MCHS, and the relative risk decreased to 4.10. Of the 226 total HGA cases in 2004–2007, 45.13% of the cases occurred in the cluster. The primary spatial cluster in 2008–2011 expanded upon the 2004–2007 cluster range to envelope the entire northern half and some eastern counties of the MCHS, with a relative risk of 4.33 and 55.82% of the total 971 HGA cases in 2008–2011 occurring in the cluster. Finally, for 2012–2016, the primary spatial cluster shifted to the eastern part of the MCHS and had a relative risk of 4.81 with 39.53% of the total 1452 HGA cases in 2012–2016 located in the cluster (Figure 6, Table 2). All detected spatial clusters were statistically significant at the α level of 0.05 and the cluster radii for the percent of the total population at risk contained within the clusters ranged from 12–28%.

**Figure 7:**
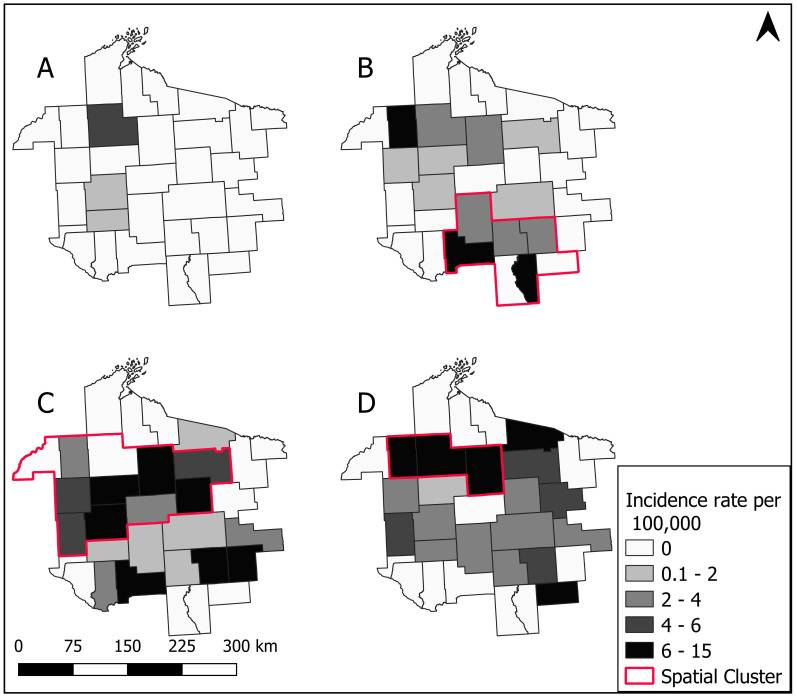
County level incidence of positive laboratory cases of babesiosis per 100,000 patients from time periods of (**A**) 2000–2003, (**B**) 2004–2007, (**C**) 2008–2011, (**D**) 2012–2016 in the Marshfield Clinic Health System. The respective primary spatial clusters are outlined in red.

Babesiosis was not analyzed for the timeframe of 2000–2003 due to a limited number of cases (*n* = 4). These first four cases of babesiosis occurred in the northwestern part of the MCHS. Analysis for babesiosis began with the 2004–2007 timeframe in which the primary spatial cluster was located in the southern expanse of the MCHS having a relative risk of 4.39 and comprising 58.62% of the total 29 babesiosis cases that occurred in the time period (Figure 7, Table 2). The 2008–2011 primary spatial cluster covered western and central counties in the MCHS and had a relative risk of 4.33, with 70.40% of the total 81 babesiosis cases occurring within the cluster. Finally, for 2012–2016, the primary spatial cluster was restricted to three counties in the northwestern part of the MCHS with a relative risk of 4.81 and containing 22.22% of the total 99 babesiosis cases (Figure 7, Table 2). All detected spatial clusters were statistically significant at the α level of 0.05 and the cluster radii for the percent of the population at risk contained within the spatial clusters ranged from 5% to 36%.

### 3.5. Sensitivity of Spatial Clusters

We explored the sensitivity of the spatial cluster analysis to narrower two-year time intervals to evaluate changes in the location and risk of disease. The time intervals used were: 2000–2001, 2002–2003, 2004–2005, 2006–2007, 2008–2009, 2010–2011, 2012–2013, 2014–2016. We found that HGA spatial clusters were robust to changes in time intervals and the spatial pattern for the original four-year interval clusters were similar to the spatial pattern of the two-year interval clusters (Figure S4). The relative risks were marginally higher using a smaller time window during the beginning of the study period but became similar during the latter half of our study period (Figure S6A).

Babesiosis showed greater sensitivity to changes in time interval aggregation between 2004–2007 and the locations of the spatial clusters for the two-year time period analysis differed slightly from the original four-year time period results (Figure S5). Relative risks of babesiosis were sensitive to changes in time aggregation in the first half of the study period when the disease was rare. Two-year time interval analyses for 2000–2001 and 2002–2003 could not be completed due to an absence of cases and low case counts (*n* = 4), respectively. Relative risks for the original analysis and sensitivity analysis became the same at the 2010–2011 two-year interval and the 2008–2011 four-year interval (Figure S6B).

### 3.6. Temporal and Seasonal Cluster Analysis

For HGA, as the temporal window increased in size, the relative risk also increased from 4.85 at the 10% temporal window to 6.61 at the 50% temporal window. No temporal clusters were identified prior to June 2010 or after August 2016 (Table 3, Figure S7). The time period of May 2011 to October 2012 was detected as part of a temporal cluster for all temporal window sizes. With babesiosis, the relative risk increased as the temporal window size decreased from 5.28 at the 50% temporal window to 6.34 at the 10% temporal window. No temporal clusters were identified prior to June 2011 or after August 2013 (Table 3, Figure S8). The time period of June 2011 to August 2012 was detected as part of a temporal cluster for all temporal window sizes.

The seasonal cluster analysis identified the months of June through August as having an elevated risk for positive laboratory test results for HGA compared to other months in the year (relative risk = 4.77, *p* value = 0.001). A total of 1610 HGA cases occurred between June and August which represented 59% of all HGA cases. The seasonal cluster for babesiosis was between the months of July to August (relative risk = 6.90, *p* value = 0.001), which represented 123 babesiosis cases (58% of all babesiosis cases).

## 4. Discussion

Using MCHS EHR data, we detected increasing risks for the tick-borne diseases, HGA, babesiosis and ehrlichiosis, in north-central Wisconsin over the last seventeen years. The location and magnitude of risk of acquiring a positive laboratory result of HGA gradually shifted east across the MCHS from 2000–2016, whereas the risk for acquiring a positive laboratory result of babesiosis shifted north and west across the MCHS from 2004–2016. Overall, the northern part of the MCHS had a greater relative risk for laboratory-positive cases of HGA and babesiosis. These findings agree with the trends in confirmed and probable cases of these diseases reported to the Wisconsin DHS for the entire state (Figure S9A) as well as reported nationally [4]. Additionally, we observed older adult males as the population with the greatest incidence of these diseases. This study provides evidence of the spread of non-Lyme tick-borne disease in the MCHS and will support the identification of at-risk areas and segments of the MCHS population.

Geographic expansion of the distribution of *I. scapularis* northward and eastward across Wisconsin was documented in past research over the same time period as our analysis [6], which may explain the changes in HGA and babesiosis risk we detected in our spatial cluster analyses. In our temporal cluster analysis, the time periods of high risk for HGA varied more with the size of the temporal window at risk compared to babesiosis. This may be due to the number of HGA cases in the MCHS, being more evenly distributed in the latter half of the study period compared to babesiosis. Changes in the reporting and introduction of a PCR diagnostic test in 2012 [40] may have influenced the temporal patterns observed for HGA which became nationally notifiable in 1999 and had a change in case definition in 2008, which required a differentiation between HGA and ehrlichiosis [23]. Laboratory-positive cases of HGA were generally more widely distributed temporally and spatially suggesting a broader distribution and higher prevalence of *A. phagocytophilum*-infected ticks than *B. microti*-infected ticks, though this is supported by limited field data [9].

For babesiosis, the temporal cluster analysis detected a rapid rise in cases in 2011 with a subsequent decline, thus resulting in the time surrounding 2011 being identified as the moment of greatest babesiosis risk. This observation may be driven in part by babesiosis becoming a nationally notifiable disease in 2011 followed by increasing awareness of the disease. The first known case of *B. microti* was detected in Wisconsin in 1985 [41] and the first laboratory-positive case in our study was in 2002. A PCR test for babesiosis was introduced at the MCHS in 2013, which is a more sensitive and accurate test compared to blood smear and serologic tests [42]. Ehrlichiosis (caused by *E. muris eauclairensis*) is a recently recognized tick-borne disease and was first identified in Wisconsin in 2009 [17] and in the MCHS in 2013 following the introduction of a PCR test capable of identifying it [40].

We found seasonal trends within the MCHS for HGA, babesiosis and ehrlichiosis to closely mirror those observed across Wisconsin through passive surveillance (Figure S9B). Our observed seasonal trends for non-Lyme tick-borne diseases supports previous research identifying summer months as generally having the most tick-borne disease cases [43,44], which is likely related to tick phenology. Past research at military bases in Minnesota, Wisconsin and Pennsylvania noted populations of *I. scapularis* nymphs peaked in June then subsequently declined [45]. It is likely that as nymph populations increase in the spring and summer along with corresponding increases in outdoor activity, that more people may be exposed to ticks and report positive laboratory results in the summer when they seek treatment. It was interesting to note that babesiosis cases showed a sharp and transient peak in cases during the summer while HGA and ehrlichiosis were more sustained throughout this same period. This observation may inform public health surveillance efforts to prepare for a rapid increase in babesiosis cases during the summer compared to a more sustained and evenly distributed case load of HGA. Both HGA and babesiosis were detected year-round in the MCHS. Detection of these diseases in the winter is most likely due to a lag in time from when an individual was exposed to a tick bite to the time when they sought treatment, given that exposure to ticks in the winter in Wisconsin is highly unlikely.

The risk of laboratory-positive cases of HGA and babesiosis showed strong spatial and temporal heterogeneities. At the beginning of our study period, high risk for HGA was identified in the northwestern part of the MCHS. The area of risk expanded eastward over time and, by the last four-year time period examined, the highest risk areas were in the easternmost region of the MCHS. This pattern was different from babesiosis, which initially had the greatest risk in the southern part of the MCHS and gradually shifted to the northwestern part of the MCHS. The spatial clusters we identified for HGA and babesiosis were typically located in different regions of the MCHS over the 4-year time periods studied, indicating that the geographic risk for these diseases may occur in more geographically discrete areas that vary over time.

Our estimated HGA and babesiosis spatial clusters within the MCHS included counties with the highest reported incidences reported to the state of Wisconsin [18,42]. Interestingly, the spatial clusters for HGA and babesiosis only overlapped in 2008–2011. It is unclear why the spatial clusters for two diseases spread by the same tick vector had disparate patterns. Research examining the infection prevalence of *I. scapularis* nymphs in Minnesota noted similar spatial patterns of *A. phagocytophilum* and *B. microti* infection prevalence across the state. However, the authors acknowledged differences in nymph infection prevalence between *A. phagocytophilum* and *B. microti* may be a result of the differences in the efficiency of the two organisms, acquired by larval ticks from their natural reservoirs [14]. The primary reservoir for human infectious *A. phagocytophilum* is the white-footed mouse [46]. Strains of *A. phagocytophilum* that are not human-infectious (the Variant 1 strains) are maintained in a lifecycle involving *I. scapularis* ticks and white-tailed deer, and deer do not become infected with the human infectious strains [47]. Conversely, Variant 1 strains are incapable of infecting white-footed mice [48]. The circulation of both human-infectious and non-infectious strains of *A. phagocytophilum* between *I. scapularis* and different reservoir hosts may produce complex ecological patterns of *A. phagocytophilum* distributions and have an impact on human disease risk. Little is known about strain competition or landscape distribution in the tick host, and this topic represents a potential area of future research. Another factor which may influence the spatial distributions of human non-Lyme tick-borne diseases is tick movement. Ticks only move short horizontal distances on their own [49] and white-tailed deer are an important host of adult *I. scapularis,* whose travel likely contributes to the geographic spread of *I. scapularis* in North America. In regions on the margins of *I. scapularis* distributions, established populations of *I. scapularis* can be found in isolated islands of forested habitat surrounded by inhospitable environments such as farmland [50]. It is likely that these populations became established by travel between habitat islands by deer or other large mammals bearing engorged female *I. scapularis*. Thus, the observed differences in spatial clustering patterns for HGA and babesiosis in our study may be the result of complex human-vector–reservoir interactions and reinforce the need to analyze tick-borne diseases separately even if they are vectored by the same organism, since each disease may present with unique spatial arrangements.

Despite the spatial variability in non-Lyme tick-borne diseases, it is important to note that the area of greatest risk for both HGA and babesiosis (regardless of time) was centered in the north central part of the MCHS. This north-central region of the MCHS typically has higher incidence rates of non-Lyme tick-borne diseases, similar to those observed for Lyme disease, compared to the rest of Wisconsin [9,42]. This region is predominantly a sparsely populated rural area which supports a higher abundance of forested habitat suitable for *I. scapularis* and presumed enzootic transmission of tick-borne diseases compared to more populated, less forested urban areas in the state [51]. Traditionally, the risk of Lyme disease in Wisconsin has been attributed to the higher risks of encountering ticks while engaging in occupational and recreational activities in heavily forested, rural areas of Wisconsin rather than to peridomestic exposure [51,52]. Now that populations of *I. scapularis* are established and humans are at risk of tick-borne diseases in nearly every county of the state, reevaluation of the activities that people are most likely engaged in while encountering ticks is needed. The Tick App project at the University of Wisconsin-Madison aims to help fill this need [53]. Social indicators such as poverty and the number of vacant housing units have been associated with increased risks of ehrlichiosis (caused by *E. chaffeensis*) and Rocky Mountain spotted fever not studied in Wisconsin, but in other places in the Midwest and Great Plains regions of the USA [54,55,56,57]. Efforts to examine the associations between these factors with the non-Lyme tick-borne diseases in our study may provide additional insight into risk factors for these diseases.

Our results provide important information describing the characteristics of individuals infected with non-Lyme tick-borne diseases in an endemic region as well as describing spatial, temporal and seasonal changes in risk. We found evidence that demographic characteristics of age- and sex-modified risk for HGA and babesiosis. This is supported by previous research in the United States that indicated that older adults and males are at a higher risk of HGA and babesiosis infections [43,44,46,58]. It is unclear whether the relationship between increasing tick-borne disease risk and age is causal or if this is an artifact of surveillance [43]. Indeed, recent serosurveys of HGA in Ontario, Canada found no significant differences in the mean ages of seropositive and seronegative patients who were tested [59], suggesting that infection in younger individuals may be under-detected at greater rates than older individuals, which may have resulted in our observation of an older mean age of laboratory-positive cases for non-Lyme tick-borne diseases in the MCHS. A retrospective study of HGA pediatric cases in the MCHS also found evidence that infections in children are clinically less severe than in adults [60], suggesting that they may be less likely to be tested for the disease than adults. These differences in detection based on age have important consequences for the data collected through surveillance and may impact the trends and observations of these diseases. Given that MCHS policies do not restrict providers from ordering tests, however, it is unlikely that the differences we detected based on age or sex are due to a system-imposed bias in the patients tested for non-Lyme tick-borne diseases by providers, though there may be differences related to clinician specialty or experience, which we are currently exploring in separate studies.

High-quality information on tick-borne disease cases is critical to public health action since tick-borne diseases may be severely underreported. For instance, Lyme disease is estimated to infect around 329,000 people annually in the USA, 10 times greater than the actual number reported to Centers for Disease Control and Prevention [61]. It is likely other non-Lyme tick-borne diseases also suffer from high levels of underreporting. A comparison of state-reported Lyme disease cases and cases identified in medical records at MCHS found evidence of significant underreporting, with only about 34% of cases reported [62]. The accuracy of reporting for HGA and babesiosis has not been systematically examined, though the Wisconsin Division of Public Health instituted an automatic electronic laboratory reporting system (which the MCHS participates in) beginning in 2007, which is expected to improve surveillance [63]. The advantages of using EHR data directly for surveillance may include a more timely identification of high-risk areas to help inform providers and the public of the current risk of tick-borne diseases. In addition, longitudinal data on subpopulations may be more easily collected and analyzed for unexpected or emerging trends. Finally, with our data we were able to estimate the percentage of patients with co-infections, as the results for each test done on patients are readily available. As more health care systems adopt electronic medical records, such integration with public health surveillance systems should only improve our understanding of the geographic and temporal trends and the characteristics of the people at greatest risk of non-Lyme tick-borne diseases. In summary, our findings may inform preventive measures to lessen the public health burden of tick-borne diseases in the MCHS and improve the case detection of these diseases.

### Limitations

The incidence rates of HGA, babesiosis and ehrlichiosis calculated in our study were based on laboratory test results in the MCHS, thus we may be overestimating the number of non-Lyme tick-borne disease cases without also reviewing clinical information and exposure history to ensure that all patients meet CSTE case definitions. For instance, serologic tests may have been false positives in some cases. However, since Wisconsin is an endemic region for non-Lyme tick-borne diseases, positive serologic test results are expected to have a high positive predicted value, such that a positive result still indicates that an individual was probably exposed to the pathogen via a tick bite at some point in time. Positive PCR and blood smear tests are indicative of active infections and tend to have lower false positive rates, such that the evidence for true infections with these types of laboratory evidence is much higher. Positive blood smears for HGA are ranked as supportive evidence in the CSTE case definition because of the concern that toxic granulation, various cytoplasmic inclusions, and staining artifacts on slides may be misinterpreted as bacterial morulae [64].

In addition, we cannot evaluate the temporal lag from when a patient was tested for a disease compared to the time when exposure to the ticks occurred. Therefore, our results represent the time at greatest risk for having a positive laboratory test, not the time of greatest risk for exposure to ticks. However, in our selection of cases, we applied strict guidelines to minimize the chance of recording an individual twice for the same infection event. Furthermore, our temporal analysis was done at a monthly interval and the results spanned multiple months to years in the seasonal and temporal cluster analyses. Even given the lag in time from tick exposure to positive laboratory testing, the length of time included in the seasonal and temporal cluster analysis results should be robust to short temporal differences.

We used the county of residence as the spatial unit of analysis which limits interpretation regarding the actual location of exposure risk, as individuals may have been infected outside this boundary. Therefore, we must interpret our spatial cluster analysis under the assumption of county-level disease risk and not risk of tick exposure. However, it is important to identify counties with higher levels of disease risk, as this can inform public health intervention strategies and resource allocation to concentrate mitigation efforts in populations with higher levels of tick-borne disease incidence.

Finally, the choice of spatial and temporal scanning windows may have impacted our observed results. Sensitivity analysis revealed that HGA and babesiosis were susceptible to changes in temporal aggregation in the spatial cluster analysis during the first half of the study period when laboratory-positive cases were sparse. However, the relative risks remained robust to scanning window sizes during the latter half of the study period when more cases were observed. Additionally, the geographic patterns of identified spatial clusters for HGA and babesiosis remained consistent following sensitivity analysis.

## 5. Conclusions

Our study found significant changes in the geographical and temporal trends of non-Lyme tick-borne diseases in north-central Wisconsin over a seventeen-year period. The evidence suggests that non-Lyme tick-borne disease cases are increasing, and the risks for the diseases expanded geographically and varied over geographic space and time. The specific regions at greatest risk tended to be distinct for HGA and babesiosis. Future research should investigate potential biological, environmental and behavioral mechanisms which may explain how and why certain areas of the MCHS had greater risk for HGA versus babesiosis than other areas and vice versa. The spatial clusters of disease identified in this research should be subjected to more intense entomological and epidemiological studies to better understand the human–vector dynamics and potentially prevent future cases of tick-borne diseases in this region. In addition, by examining medical record data directly, variation due to provider practices and other factors related to the delivery of health care may be incorporated into models developed to predict risk.

## Figures and Tables

**Table 1:** Characteristics of non-Lyme tick-borne disease cases and laboratory confirmation ^1^ counts among the Marshfield Clinic Health Care System (MCHS) population: 2000–2016.

Disease by Type & Lab Test	Total	Age at Positive Lab Result (Years)	Age Range ^2^ (Years)	Sex	
	*N* (% Total)	Mean (SD)		Male (%)	Female (%)
**HGA ^3^**	2728 (92.3%)	54 (20)	1 to >89	1480 (54.3%)	1248 (45.7%)
**Confirmed:**	809	59 (18)	2 to >89	480 (59.3%)	329 (40.7%)
PCR +	665	60 (18)	2 to >89	390 (58.6%)	275 (41.4%)
IFA 4-fold rise	144	56 (17)	4 to >89	90 (62.5%)	54 (37.5%)
**Supportive:**	1919	51 (20)	1 to >89	1000 (52.1%)	919 (47.9%)
Smear +	109	62 (17)	4 to >89	66 (60.6%)	43 (39.4%)
IFA +	1810	51 (20)	1 to >89	934 (51.6%)	876 (48.4%)
**Babesiosis**	213 (7.2%)	59 (16)	7 to >89	134 (62.9%)	79 (37.1%)
**Confirmed:**	98	62 (15)	18 to >89	68 (69.4%)	30 (30.6%)
PCR +	55	60 (15)	18 to >89	36 (65.5%)	19 (34.5%)
Smear +	26	65 (15)	29 to 86	19 (73.1%)	7 (26.9%)
PCR+ & Smear +	17	63 (16)	28 to 85	13 (76.4%)	4 (23.6%)
**Supportive:** IFA +	115	56 (17)	7 to >89	66 (57.4%	49 (42.6%)
**Ehrlichiosis ^4^**	15 (<1%)	69 (8)	60 to 83	6 (40.0%)	9 (60.0%)
**All Diseases Combined**	2956 (100%)	54 (20)	1 to >89	1620 (54.8%)	1336 (45.2%)

^1^ Positive tests confirmation. PCR = polymerase chain reaction; IFA = indirect immunofluorescence assay; smear = blood smear. ^2^ Where age is rounded to nearest year. Max ages over 89 years are suppressed for privacy. ^3^ Where human granulocytic anaplasmosis (HGA) represents cases caused by *A. phagocytophilum*, babesiosis represents cases caused by *B. microti* and ehrlichiosis represents cases caused by *E. muris eauclairensis.* ^4^ All ehrlichiosis cases were laboratory confirmed using PCR.

**Table 2:** Summary of non-Lyme tick-borne disease risk within spatial clusters across north central Wisconsin stratified by years.

Tick-Borne Disease	Years	Relative Risk of Disease ^1^	Cluster Size (% Population at Risk)	*p*-Value	Observed Cases in Cluster	Expected Cases in Cluster
**HGA**	2000–2003	11.85	12.70%	<0.001	50	10.04
	2004–2007	4.10	16.70%	<0.001	102	37.74
	2008–2011	3.30	27.68%	<0.001	542	268.78
	2012–2016	3.88	14.41%	<0.001	574	209.30
	Full Period	3.00	27.54%	<0.001	1454	751.34
**Babesiosis ^2^**	2000–2003	-	-	-	-	-
	2004–2007	4.39	24.41%	0.013	17	7.08
	2008–2011	4.33	35.44%	<0.001	57	28.71
	2012–2016	4.81	0.06%	<0.001	22	5.55
	Full Period	3.90	0.05%	<0.001	38	11.24
**Combined Disease ^3^**	Full Period	2.92	27.54%	<0.001	1554	814.14

^1^ Denotes the risk of a positive lab result for a tick-borne disease for individuals residing within a cluster compared to individuals residing in counties outside a cluster. ^2^ There were insufficient babesiosis cases (*n* = 4) during the 2000–2003 timeframe to perform a clustering assessment. ^3^ Combined disease includes positive laboratory cases of human granulocytic anaplasmosis (HGA), babesiosis and ehrlichiosis.

**Table 3:** Temporal clusters identified for laboratory positive cases of non-Lyme tick-borne diseases by disease type over varying time windows (10, 20, 30, 40 and 50%) with relative risks.

Tick-Borne Disease	Timeframe ^1^	Scanning Window ^2^	Relative Risk ^3^	*p* Value	Observed Cases	Expected Cases
**HGA**	05/2011–10/2012	10%	4.85	0.001	898	250.48
	05/2011–08/2014	20%	5.07	0.001	1512	537.41
	06/2010–09/2014	30%	5.51	0.001	1799	709.29
	06/2010–08/2016	40%	6.61	0.001	2163	1000.92
	06/2010–08/2016	50%	6.61	0.001	2163	1000.92
**Babesiosis**	06/2011–08/2012	10%	6.34	0.001	73	16.19
	06/2011–08/2013	20%	5.28	0.001	96	28.67
	06/2011–08/2013	30%	5.28	0.001	96	28.67
	06/2011–08/2013	40%	5.28	0.001	96	28.67
	06/2011–08/2013	50%	5.28	0.001	96	28.67

^1^ Denotes the time frame in which the temporal cluster was identified. ^2^ Denotes the percentage of the total study time period the temporal cluster was allowed to be in size. ^3^ Denotes the risk of a positive laboratory case of a non-Lyme tick-borne disease with residing within the specified moment in time compared to cases residing outside of that moment in time.

## Supplementary Materials

The following are available online at https://www.mdpi.com/1660-4601/17/14/5105/s1, Figure S1: (**A**) MCHS population as a percentage of the Wisconsin 2010 census population for the counties within the MCHS area and (**B**) MCHS population stratified by broad age categories, 2000–2016. Figure S2: Systematic geocoding and case identification workflow applied for MCHS non-Lyme tick-borne disease cases, 2000–2016. Figure S3: Annual mean age of laboratory positive cases for (**A**) HGA, (**B**) babesiosis and (**C**) ehrlichiosis in the MCHS from 2000–2016 stratified on sex. Counts of laboratory positive cases for (**D**) HGA, (**E**) babesiosis and (**F**) ehrlichiosis in the MCHS from 2000–2016 stratified on sex. Figure S4: Primary spatial clusters of HGA within the MCHS for two-year time intervals from 2000–2016. Figure S5: Primary spatial clusters of babesiosis within the MCHS for two-year time intervals from 2000–2016. Figure S6: Relative risk of acquiring a positive laboratory result for a non-Lyme tick-borne disease in the MCHS from 2000–2016, comparing the original 4-year time intervals results to 2-year time intervals for sensitivity analysis. Figure S7: Temporal clusters of laboratory positive cases of HGA using varying time windows (10, 20, 30, 40 & 50%) in the MCHS from 2000–2016. Figure S8: Temporal clusters of laboratory positive cases of babesiosis using varying time windows (10, 20, 30, 40 & 50%) in the MCHS from 2000–2016. Figure S9: Confirmed and probable cases of non-Lyme tick-borne diseases reported to the Wisconsin DHS from 2000–2016.
