# Supplementary material for: Spatio-Temporal Dynamics of Tick-Borne Diseases in North-Central Wisconsin from 2000–2016
Source: Int J Environ Res Public Health. 2020 Jul 15;17(14):5105. doi: 10.3390/ijerph17145105 (PMC7400118; doi:10.3390/ijerph17145105)
Supplement: Supplementary file 1 [file ijerph-17-05105-s001.pdf]

International Journal of
Environmental Research and Public Health

Supplementary Online Content

Spatio-temporal dynamics of tick-borne diseases in north-central Wisconsin from 2000 – 2016

Table of Contents

Figure S1. A) MCHS population as a percentage of the Wisconsin 2010 census population for the counties within the MCHS area and B) MCHS population stratified by broad age categories, 2000 – 2016.
Figure S2. Systematic geocoding and case identification workflow applied for MCHS non-Lyme tick-borne disease cases, 2000 – 2016.
Figure S3. Annual mean age of laboratory positive cases for A) HGA, B) babesiosis and C) ehrlichiosis in the MCHS from 2000 – 2016 stratified on sex. Counts of laboratory positive cases for D) HGA, E) babesiosis and F) ehrlichiosis in the MCHS from 2000 – 2016 stratified on sex.
Figure S4. Primary spatial clusters of HGA within the MCHS for two-year time intervals from 2000 - 2016.
Figure S5. Primary spatial clusters of babesiosis within the MCHS for two-year time intervals from 2000 - 2016.
Figure S6. Relative risk of acquiring a positive laboratory result for a non-Lyme tick-borne disease in the MCHS from 2000 - 2016, comparing the original 4-year time intervals results to 2-year time intervals for sensitivity analysis.
Figure S7. Temporal clusters of laboratory positive cases of HGA using varying time windows (10, 20, 30, 40 & 50%) in the MCHS from 2000 – 2016.
Figure S8. Temporal clusters of laboratory positive cases of babesiosis using varying time windows (10, 20, 30, 40 & 50%) in the MCHS from 2000 – 2016.
Figure S9. Confirmed and probable cases of non-Lyme tick-borne diseases reported to the Wisconsin DHS from 2000 – 2016.

This supplementary material has been provided by the authors to give readers additional information about their work.

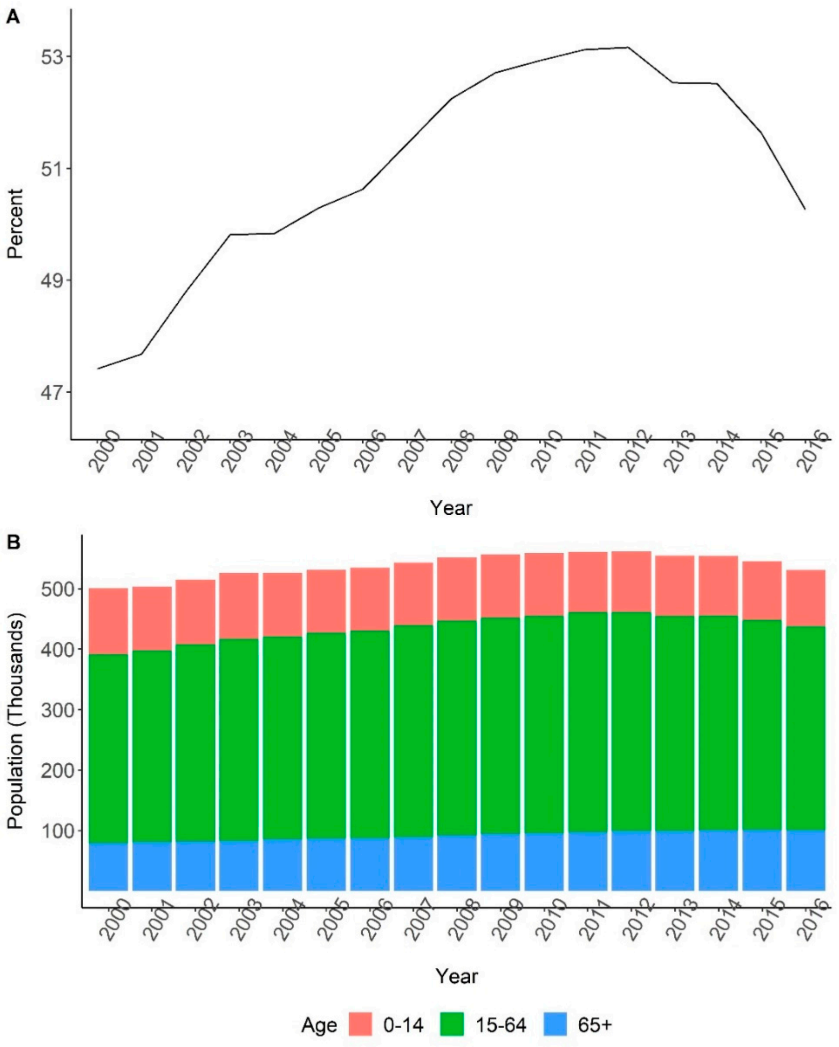

Figure 1. A) MCHS population as a percentage of the Wisconsin 2010 census population for the counties within the MCHS area and B) MCHS population stratified by broad age categories, 2000 – 2016.

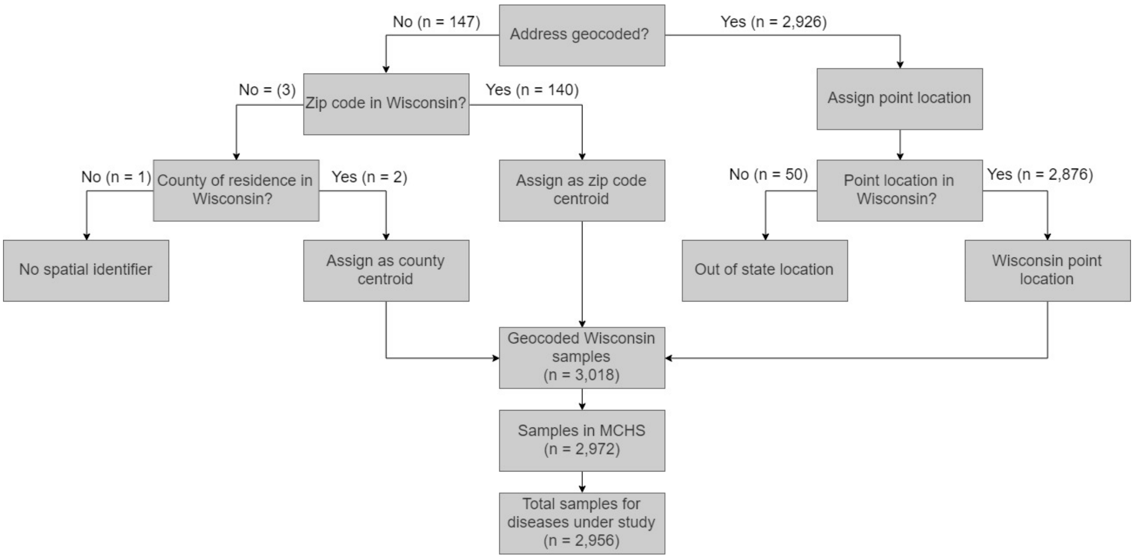

Figure 2. Systematic geocoding and case identification workflow applied for MCHS non-Lyme tick-borne disease cases, 2000 – 2016.

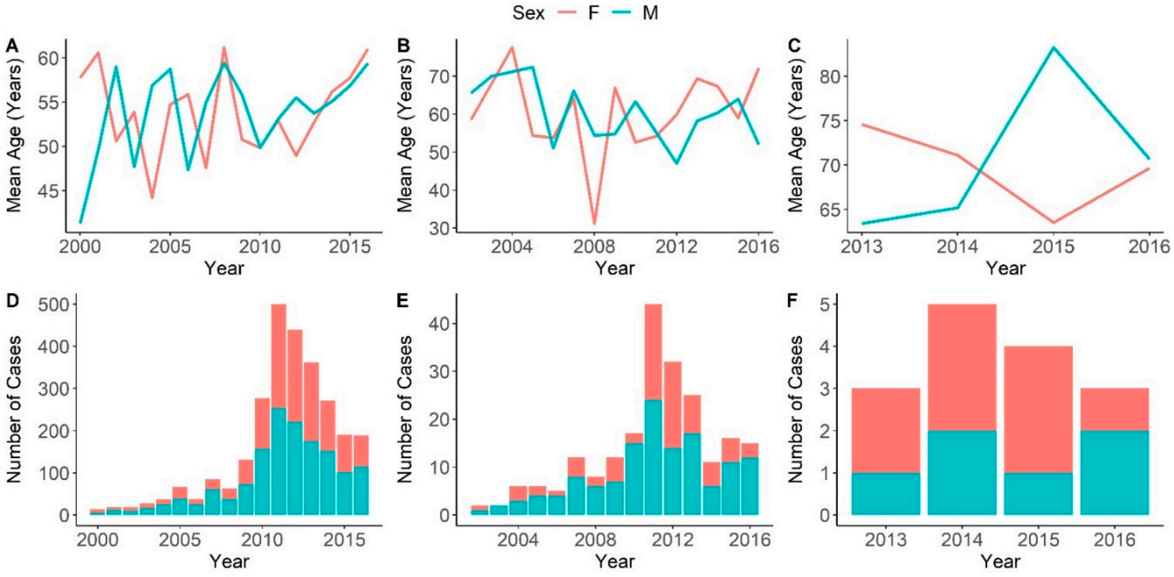

Figure 3. Annual mean age of laboratory positive cases for A) HGA, B) babesiosis and C) ehrlichiosis in the MCHS from 2000 – 2016 stratified on sex. Counts of laboratory positive cases for D) HGA, E) babesiosis and F) ehrlichiosis in the MCHS from 2000 – 2016 stratified on sex.

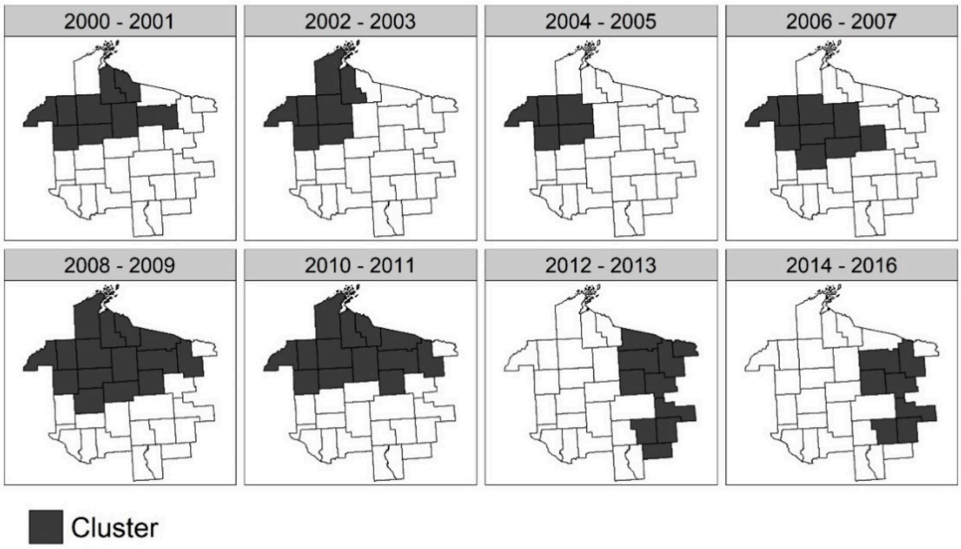

Figure 4. Primary spatial clusters[1] of HGA within the MCHS for two-year time intervals from 2000 - 2016. [1] All detected spatial clusters significant at the α level of 0.05.

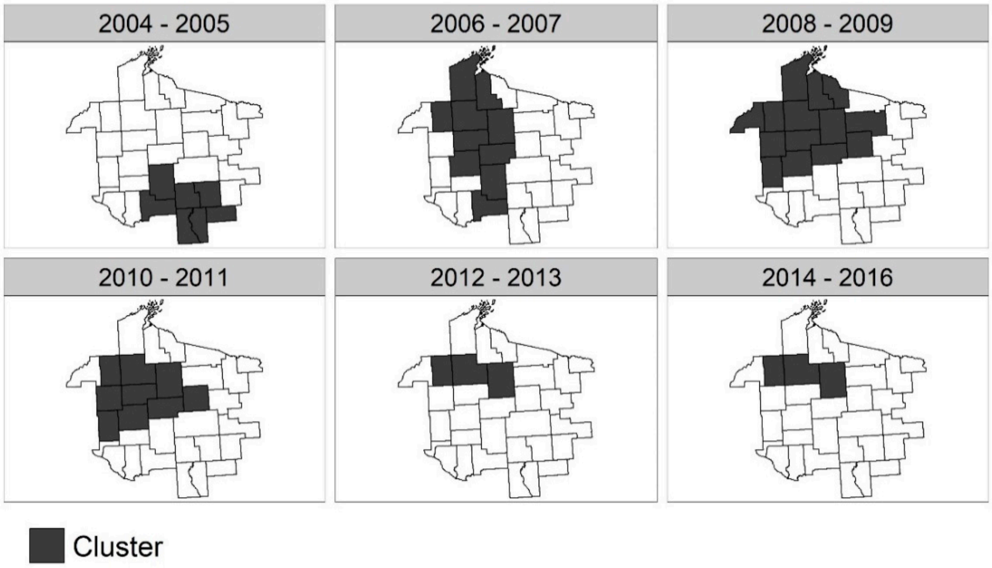

Figure 5. Primary spatial clusters[1] of babesiosis within the MCHS for two-year time intervals from 2000 – 2016. [1] All detected spatial clusters, except for 2014 – 2016, significant at the α level of 0.05.

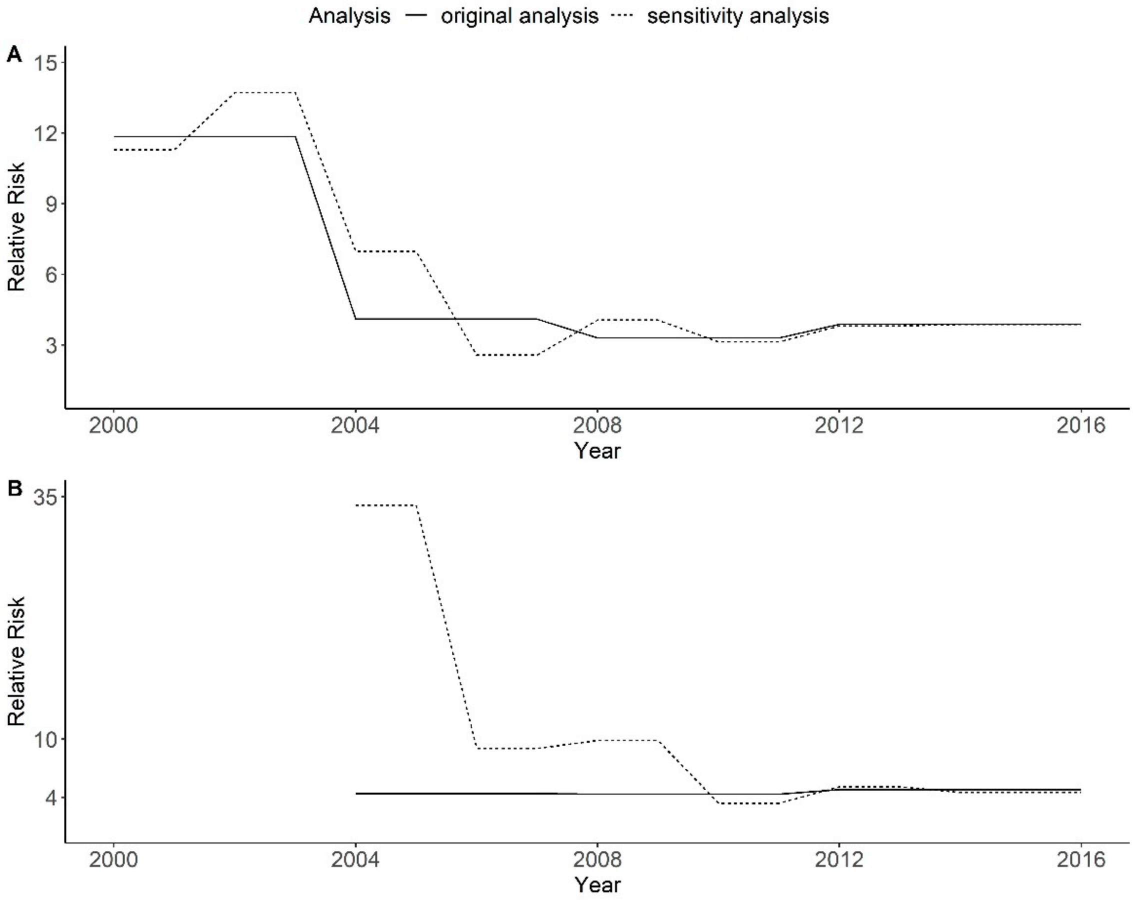

Figure 6. Relative risk of acquiring a positive laboratory result for a non-Lyme tick-borne disease in the MCHS from 2000 - 2016, comparing the original 4-year time intervals results to 2-year time intervals for sensitivity analysis. A) Relative risk of acquiring a positive laboratory result for HGA, comparing the original analysis results to the sensitivity analysis results. B) Relative risk of acquiring a positive laboratory result for babesiosis, comparing the original analysis results to the sensitivity analysis results.

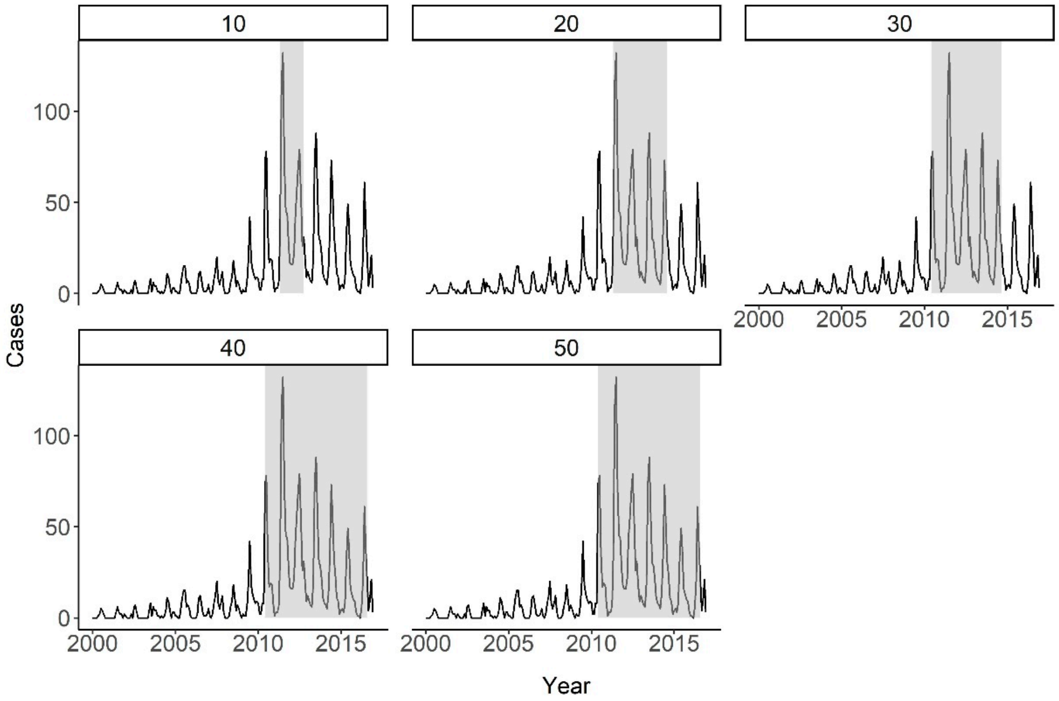

Figure 7. Temporal clusters of laboratory positive cases of HGA using varying time windows (10, 20, 30, 40 & 50%) in the MCHS from 2000 – 2016.

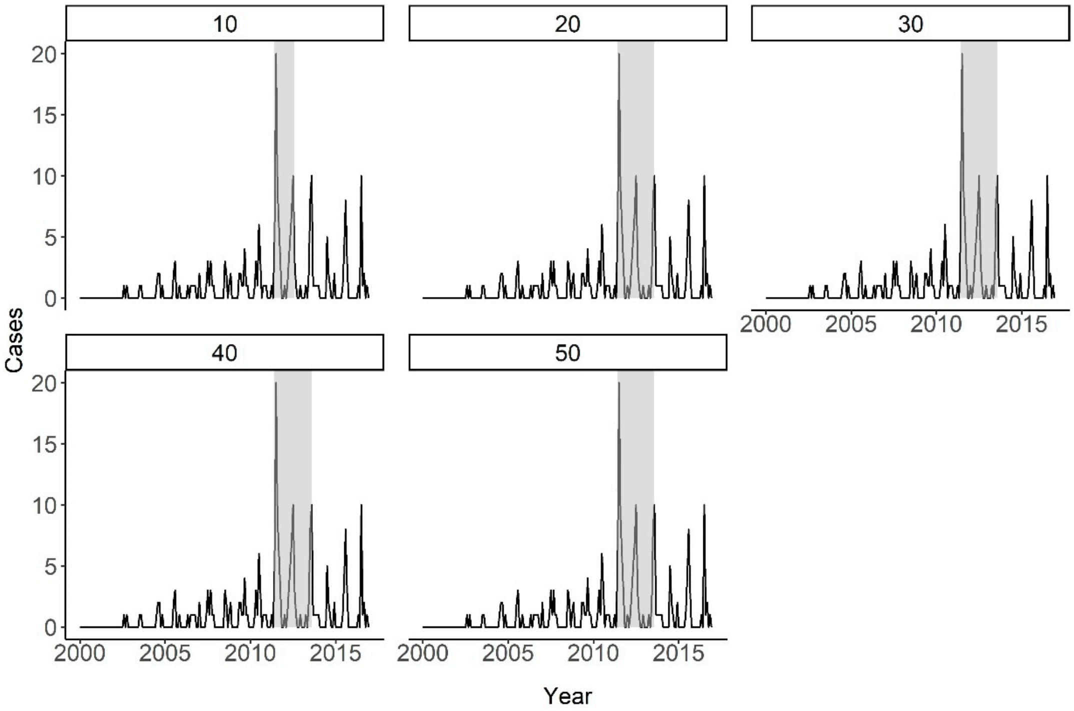

Figure 8. Temporal clusters of laboratory positive cases of babesiosis using varying time windows (10, 20, 30, 40 & 50%) in the MCHS from 2000 – 2016.

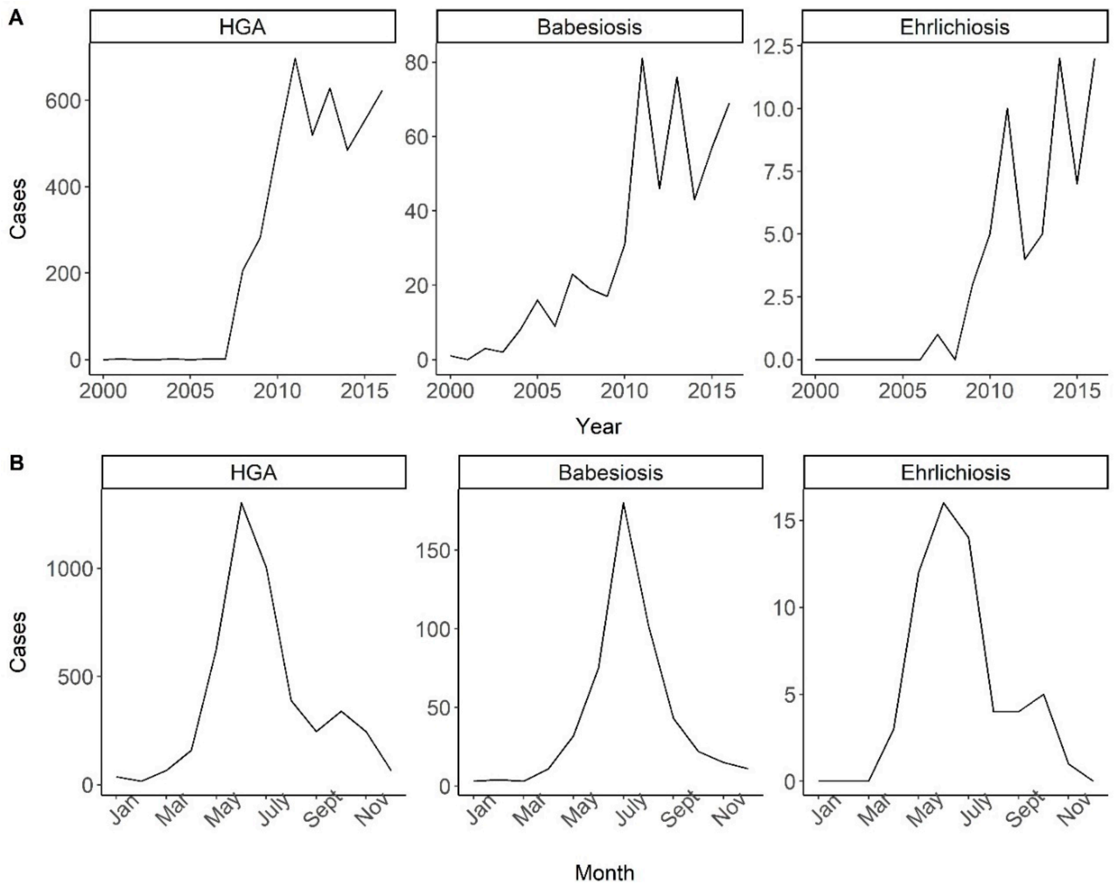

Figure 9. Confirmed and probable cases of non-Lyme tick-borne diseases reported to the Wisconsin DHS from 2000 – 2016. Annual HGA, babesiosis, and ehrlichiosis cases (confirmed and probable), and B) Seasonal HGA, babesiosis, and ehrlichiosis cases (confirmed and probable). Ehrlichiosis cases represent those caused by E. muris eauclairensis. (Source: Wisconsin DHS, 2020).
